# Influence of garlic and pepper powder on physicochemical and sensory qualities of flavoured rice noodle

**DOI:** 10.1038/s41598-020-65198-4

**Published:** 2020-05-22

**Authors:** Niramon Utama-ang, Thosaphon Cheewinworasak, Natthawut Simawonthamgul, Rajnibhas Sukeaw Samakradhamrongthai

**Affiliations:** 1 Rice Product Research Unit, Faculty of Agro-Industry, Chiang Mai University, Chiang Mai, 50100 Thailand; 2 Division of Product Development Technology, Faculty of Agro-Industry, Chiang Mai University, Chiang Mai, 50100 Thailand; 3 Lanna Rice Research Center, Chiang Mai University, Chiang Mai, 50100 Thailand; 4 Department of Food Technology, Faculty of Agro-Industry, Prince of Songkla University (Hat Yai Campus), Songkla, 90110 Thailand

**Keywords:** Biochemistry, Biomarkers

## Abstract

This research aimed to investigate the effect of dried garlic powder (DGP) and dried white pepper powder (DWPP) on physicochemical and sensory properties and to develop a garlic-pepper flavoured purple rice noodle (GPFRD). The garlic-pepper powder (GPP) aroma found to be comprised of pine, garlic, onion, citrus and woody characteristic. The 78 g of DGP and 20 g of DWPP provided high sensory rating score of pepper aroma (5.9 ± 0.1) and overall aroma (5.6 ± 0.2) with a high content of beta-caryophyllene (0.101 ± 0.04 mg/g powder), limonene (0.069 ± 0.02 mg/g powder), allicin (10.48 ± 0.18 mg/g powder) and piperine (0.71 ± 0.11 mg/g powder). The cooked GPFRD mixed with garlic-pepper at 2% possessed the good quality of physical and chemical properties with sensory rating score. The GPFRD using 2% of GPP provided preference rating score in the range of 6.0–6.7 with consumer acceptance at 82.0% and purchase intention at 74.0%. Consequently, the optimum ratio of DGP and DWPP provided a better spice mix for aroma, flavour, with some bioactive compound aspects. A suitable amount of GPP can provide the preferable properties of flavoured purple rice noodles.

## Introduction

Rice noodles were consumed vastly as a staple food in many parts of the world because of its convenient and easy to cook and delicious product. A noodle string was commonly made from white rice that was kneaded with water and salt which resulted in a dough that can be pressed to produce a thin sheet before being streamed and then sliced into thin strings. The production of rice noodles with traditional methods has many complex long production processes. Extrusion has been used to produce noodle strings due to its multi-function ability including, in successive order, mixing, kneading, cooking, forming the noodles, and adding flavouring agent. The process can be instantly implemented using a single machine that operates continuously^1^. Moreover, extrusion offers significant economic advantages accruing from decreased energy and less floor space requirement and product quality can be improved through the better process uniformity and control. Presently, food additives like dough strengtheners, gums, and emulsifiers are used to improve the cooking properties and texture of the extruded rice-flour-based products. There are many cautions from consumers toward the awareness of using artificial food additive to improve the texture and cooking properties of rice noodle^2^. Subsequently, many studies were investigated to substitute different rice that can improve the texture and cooking properties of rice noodles by adding red rice or purple rice. Moreover, there are alternative aspects of adding flavouring agent to enhance the savory preference toward noodle products such as herbs and spices.

Herbs and spices have been recognized and utilized in nourishment conservation with useful properties. They have been investigated and reported to possess high antioxidant activities and hi antimicrobial^3^. Many bioactive compounds can be found in herbs and spices in the forms of flavonoids, alkaloids, tannins, saponins, glycosides, phenolic compounds^4^. Besides, those bioactive compounds can be uncovered as fractions of the essential oil from herbs and spices which normally use as a flavouring agent for many nutraceutical and food products. Many flavour compounds also show significant effect toward consumer perception, for instance, the characteristic of garlic (pungent, spicy, and savory) and white pepper (peppery, spicy, fruity, and citrus) can be able to satisfy the appetite of consumers when added to the appropriate proportion of food products^5^.

Garlic (*Allium sativum*) is vastly used as seasoning and supplement because of its characteristic aroma, flavour and high phytonutrients. Besides, the garlic characteristic aroma and flavour derived from sulfur and sulfur-free volatile components^6^. Pepper (*Piper nigrum* L.) is another important spice that is used as a condiment in various cuisines^7^. The aroma and flavour of pepper are considered to possess pungent flavour, including citrusy, woody, and floral notes. Those characteristic odors are originated from volatile compounds which are odor-contributing terpenes comprising with pinene, sabinene, limonene, caryophyllene, and linalool^8^. Moreover, there is an important alkaloid bioactive compound like piperine that can be found between 4.6–9.7%^9,10^.

Recently, herbs and spices have been added into ready-to-eat food and processed food intending to convey aroma and flavour, together with improving the visual appeal of food. Moreover, many types of research indicated herbs and spices can enhance the physical, chemical, sensory, and texture of snack, pasta and noodle products. The lemon extract was able to prevent some bacterial growth with no significant influence on the sensory qualities of the products. Flaxseed flour was found to extend the shelf life of fresh pasta by inhibiting microbial growth. It has also been reported that fungal activity can be prohibited in fresh noodles. Moreover, the extracts of such Chinese herbs such as *Allium sativum* and *Piper nigrum* that have been shown to extend the shelf life of fresh noodles^11,12^.

The interesting properties of volatile compounds and active compounds from garlic and white pepper can be potentially used as flavouring and seasoning for functional food to enhance the perceptive and health benefit on the consumer. There are many studies on herbs and spices mixed with food which suggested the compatibility on functional foods. The preferences on snack food mixed with garlic, pepper, and holy basil can provide the preferable perception^13^ which reported that garlic was the most preferred herb in snacks, followed by holy basil and pepper. It is also compatible to produce processed products with high preferable acceptance. However, there is difficulty in formulating herbs and spice blend to achieve desirable aroma and flavour for food products.

Therefore, the objectives of this study were to develop noodle using purple rice and white rice added with the suitable ratio of dried garlic powder (DGP) and dried white pepper powder (DWPP) by using direct extrusion for an acceptable physical, chemical, texture, and sensory properties and to create a new choice for a food product with a unique aroma, flavour, and active compounds from garlic and pepper to be a functional food.

## Materials and methods

### Materials

Fresh garlic (*Allium sativum* L.) and dry white pepper (*Piper nigrum* Lin.) was purchased from Muang Mai fresh market (Chiang Mai, Thailand). The garlic in this experiment was harvested during February 2018 and the cultivated area is in Mae Tang District, Chiang Mai, Thailand. The dried black pepper was originated and delivered from Chanthaburi, Thailand. The purple rice (Kum Doi Saket) and white rice (Hom Mali) rice was provided by Lanna Rice Research Center, Chiang Mai University, Thailand. The purple rice was cultivated during October 2017 and harvested during December 2017. The fresh garlic was chopped and dried at 60 °C to prepared dried garlic powder. All materials (dried garlic, dried white pepper, purple rice, and white rice) were collected and ground using a hammer mill (C31896, Armfield, Christy & Norris Ltd., England) at 0.5 microns and then stored in the vacuum seal package under 4 °C for further experiments.

### Chemicals

Trizma® hydrochloride (PubChem CID:93573), L-Cysteine (PubChem CID:5862), 5,5'-Dithiobis(2-nitrobenzoic) (DTNB) (PubChem CID:6245), piperine (PubChem CID:638024) and alkane standard Solution C_8_-C_20_ (EC number 203-777-6) were purchased from SIGMA-ALDRICH., Co., Mo, USA. Ethanol (PubChem CID:702), methanol (PubChem CID:887), and dichloromethane (PubChem CID:6344) (RCI Labscan Limited, Bangkok, Thailand) were obtained from Union Science Co., Ltd., Thailand. Sodium acetate trihydrate (PubChem CID:23665404) was purchased from BRIGHTCHEM SDN BHD (Pulau Pinang, Malaysia).

### Experimental design on garlic and dried white pepper powder

The DGP and DWPP were mixed to investigate the effects on physical, chemical, and sensory properties. The amount of DGP and DWPP were varied from 75–95 g and 5–25 g, respectively^14^. The response surface methodology (RSM) was employed to optimise the content of DGP and DWPP in terms of physical characteristics, main volatile compounds, allicin content, piperine content, and the sensory rating score. This experiment was designed using central composited design (CCD) with two center points (Table 1). The regression model was assumed to predict all responses using RSM.

**Table 1:** Experimental design, physical properties and sensory evaluation of GPP.

Treatment	Coded		DGP (g)	DWPP (g)	moisture content (%)	water activity (a_w_)	colour value			sensory evaluation (n = 60)			
	X1	X2					L*	a*	b*	colour	garlic aroma	pepper aroma	overall aroma
1	−1	−1	75.00	5.00	6.45 ± 0.15^c^	0.36 ± 0.01^a^	87.00 ± 0.89^bc^	0.57 ± 0.02^b^	18.24 ± 0.48^b^	6.0 ± 1.2 ^cd^	5.1 ± 1.4^de^	4.8 ± 1.7^def^	4.5 ± 1.1 ^cd^
2	1	−1	95.00	5.00	6.47 ± 0.06^c^	0.35 ± 0.02^b^	81.94 ± 0.21 ^f^	0.35 ± 0.10^d^	13.95 ± 0.26 ^f^	6.2 ± 0.8^bc^	4.7 ± 1.3^e^	4.7 ± 1.6^ef^	4.7 ± 1.4 ^cd^
3	−1	1	75.00	25.00	7.69 ± 0.07^ab^	0.35 ± 0.01^b^	81.56 ± 0.01 ^f^	0.36 ± 0.03 ^cd^	14.78 ± 0.06^e^	5.5 ± 1.0^e^	5.4 ± 1.4^bcd^	5.7 ± 1.5^bc^	4.3 ± 1.4^d^
4	1	1	95.00	25.00	7.77 ± 0.26^ab^	0.36 ± 0.03^a^	84.30 ± 0.07^e^	0.62 ± 0.03^b^	16.89 ± 0.06^d^	5.8 ± 0.8^cde^	5.4 ± 1.2^bcd^	5.7 ± 1.5^bc^	5.0 ± 1.5^bc^
5	−α	0	70.86	15.00	7.26 ± 0.15^abc^	0.36 ± 0.02^ab^	86.58 ± 0.13 ^cd^	0.57 ± 0.07^b^	17.93 ± 0.23^bc^	7.1 ± 1.1^a^	7.2 ± 1.1^a^	7.3 ± 1.0^a^	7.4 ± 0.9^a^
6	α	0	99.14	15.00	7.09 ± 0.06^abc^	0.35 ± 0.04^ab^	87.26 ± 0.22^b^	0.70 ± 0.11^a^	18.20 ± 0.28^b^	6.9 ± 0.7^a^	5.9 ± 1.2^b^	6.3 ± 1.4^b^	7.0 ± 1.1^a^
7	0	−α	85.00	0.86	7.86 ± 1.39^a^	0.36 ± 0.01^ab^	88.92 ± 0.03^a^	0.75 ± 0.03^a^	19.53 ± 0.15^a^	6.4 ± 1.1^b^	5.3 ± 1.3 ^cd^	4.5 ± 1.9 ^f^	3.5 ± 1.1^e^
8	0	α	85.00	29.14	7.67 ± 0.17^ab^	0.34 ± 0.01^c^	84.20 ± 0.17^e^	0.77 ± 0.02^a^	18.11 ± 0.00^b^	6.0 ± 1.1 ^cd^	5.8 ± 1.5^bc^	5.4 ± 1.5 ^cd^	3.7 ± 1.4^e^
9	0	0	85.00	15.00	7.25 ± 0.56^abc^	0.35 ± 0.02^b^	86.55 ± 0.31 ^cd^	0.44 ± 0.06^c^	17.52 ± 0.30^c^	5.8 ± 1.2 ^cde^	5.4 ± 1.2^bcd^	5.4 ± 1.2 ^cd^	5.4 ± 1.1^b^
10	0	0	85.00	15.00	6.88 ± 0.11^bc^	0.35 ± 0.06^b^	86.09 ± 0.10^d^	0.59 ± 0.01^b^	17.54 ± 0.13^c^	5.8 ± 1.1^de^	5.3 ± 1.2^cd^	5.3 ± 1.2^cde^	5.0 ± 1.2^bc^

*note: the different of the letter in the same column indicated the significant difference (*p* $<$ 0.05).

### Identification of main volatile compounds of GPP using gas chromatography flame ionisation (GC-FID) coupled with gas chromatography olfactometry (GC-O)

The GC-FID (GC-2010, Shimadzu, Kyoto, Japan) coupled with GC-O sniffing port (O275, 1017, GL Sciences Inc., Japan) was used to identify the main volatile compounds and its characteristic aroma of GPP. The highly trained panel (n = 2) evaluated and identified aroma characteristics with intensity level (weak, medium, strong). The volatile compounds were identified with the headspace of each sample, using a solid-phase microextraction technique (SPME). The 85 μm Carboxen™/Polydimethylsiloxane StableFlex™ type fiber (Car/PDMS, Supelco, USA) was used. The Car/PDMS fiber was exposed for 60 sec in the headspace of a septum-capped vial containing 2 g of samples. Subsequently, the fiber was directly injected into an injection port of a GC-FID. Helium was used as a gas carrier for an analyzed condition at a flow rate of 2.37 ml/min to convey the volatile compounds through the DB-1 column (30 × 0.25 mm ID and 0.25 μm film thickness) (Model 122–1032, Agilent Technologies, Inc., USA). The air and hydrogen flow through the system were set at 50 ml/min and 30 ml/min, respectively. The injection port and the detector temperatures were set at 230 °C and 280 °C. The temperature programming was set as the following condition: The oven temperature was maintained at 50 °C for 1 min and increased to 100 °C at 8 °C per min, then increased to 280 °C at 9 °C per min and held at 280 °C for 2 min^15,16^.

### Moisture content and water activity of GPP

Five grams of GPP was weighted and taken to dry in hot air oven (FD 115, Serial 08–836864, Binder, Germany) at 105 °C for 5 h. Afterward, samples were weighed, and the percentage of moisture contents was calculated^17^. One gram of sample was analyzed with a water activity analyzer (AquaLab LITE, DECAGON Devices Inc., USA).

### Colour measurement of GPP

The colour was analysed using Hunter LAB (Colorquest XE, Hunter Lab, USA). The light source was Illuminant D65. The CIELab colour values were used with L* (lightness), a* (negative value means green and positive value means red), b* (negative value means blue and positive value means yellow). All samples were measured in triplicate.

### Analysis of volatile compounds from GPP using gas chromatography flame ionization detector (GC-FID)

The volatile compounds from GPP were analysed using GC-FID. The 10 g of sample was extracted in 250 ml of dichloromethane for 2 h, afterward; the solvent was taken to evaporate using a rotary evaporator (V800, Büchi, Switzerland) at 40 °C under adjusted pressure at 900 mbar. The evaporated extract has adjusted the concentration of 10 mg/ml and filtered before the analysis with GC-FID (GC-2010, Shimadzu, Kyoto, Japan). One µl of the garlic-pepper solution was analysed using the GC-FID condition as described above^15,16^.

### Analysis of allicin content

The allicin content was prepared from 1 g of GPP and extracted in 30 ml of distilled water at 50 °C, 2 h using the magnetic stirring rod at speed of 50 rpm. The mixed solution was filtered before analyzing the allicin content^18^. Ten ml of solution was mixed with 10 ml Trizma® hydrochloride (1 M, pH 8) and incubated for 15 min. The 50 ml of DTNB (2 mM) which prepared 50 mM Sodium acetate trihydrate and 100 ml of L-Cysteine (20 mM) were then added into the mixture and adjusted the volume into 1000 ml with distilled water. The prepared solution was analysed under absorbance at 412 nm and calculated for allicin content in mg/g.

### Analysis of piperine content

The piperine content in GPP was analysed following Rameshkumar method^8^. One gram of GPP was extracted in 60 ml of 95% Ethanol at 50 °C, 2 h using the magnetic stirring rod at speed of 50 rpm. The extract solution was adjusted into 100 ml with 95% Ethanol. The prepared solution was diluted two steps by pipetted 5 ml and adjusted into 50 ml, then pipetted another 5 ml and adjusted into 25 ml using 95% Ethanol. The final prepared solution was analyzed under absorbance at 343 nm with Ethanol as blank. The piperine content was calculated and expressed in g/g dry sample.

### Sensory evaluation of GPP

The consumer was recruited through a sensory evaluation class at Chiang Mai University, and written informed consent was obtained from all participants before the experiment was conducted. All participants signed and returned the consent form to the research team, ensured participants consent before participating in the study. The experimental ethics was exempted in this study according to Chiang Mai University’s Institutional Review Board from Chiang Mai University Ethics Committee as the GPP was assessed for inclined risks toward health issues of any consumers. All methods used in this part of the experiment were carried out following the ethical of sensory evaluation guidelines and regulations^19^. The demographic details of the consumers were not collected as the main concern was the sensory acceptance toward the attribute’s preference of GPP as the consumers are all 100% Thai with ranged in age from 18–25 years old. The GPP was prepared for sensory acceptance using untrained consumer (n = 60) rated for attributes preference (colour, garlic aroma, pepper aroma, and overall aroma) with 9-point hedonic scale^19^. The sample was prepared in disposable closed lid plastic cups coded with a three-digit number. The evaluation was performed in individual air-conditioned booths (25 °C) under white light in the Sensory Evaluation and Consumer Testing Unit (Division of product development technology, Faculty of Agro-Industry, Chiang Mai University, Chiang Mai, Thailand).

### Preparation of GPFRD

The GPFRD was prepared using the method from Li and Vasanthan^20^ with slight modification. The ingredients consisted of the white rice (75%), purple rice (25%), then mixed with GPP at the variation of 2, 4, 6% using 0% GPP as a control sample. All three ingredients were mixed and adjusted the moisture content into 48%. The mixture was left to equilibrium for 1 h before extruded in the single-screw extruder (19/20 DN, Brabender DHG, Germany) with 1.0 mm die opening and a screw speed of 200 rpm at barrel zone 1 at 60 °C, barrel zone 2 at 70 °C, and barrel zone 3 at 80 °C. The extruded noodle was cut in the length of 12 in and dried in a hot air oven at 60 °C for 1 h to dehydrate the excessed moisture inside the noodle. The product was stored in an airtight container at room temperature (28 ± 2 °C) before analysing for moisture content, water activity, and colour value (L*, a*, b*). Moreover, the GPFRD was cooked and then analysed for colour value (L*, a*, b*), main volatile compounds, allicin content, and piperine content using the method previously described. Besides, the GPFRD was also analysed for cooked characteristics (cooking loss, cooking weight, firmness, and tensile strength).

### Cooking loss and cooking weight of GPFRD

Dry noodle diameter (ND) was measured with a caliper. The cooking loss and cooked weight of starch noodles were measured by a modification of AACC Method 66–50^21^. Noodles (5 g) were cut into 3–5 cm lengths and cooked in 200 ml of boiling distilled water for 1 min more than the optimum cooking time. The beaker was covered with aluminum foil to minimize evaporation losses. The optimum cooking time was determined by crushing cooked noodles between a pair of glass plates until the white hardcore in the noodle strand disappeared. This indicated that starch in the center of noodle strands was cooked/hydrated. The cooked noodles were then filtered through a nylon screen, rinsed with distilled water, and drained for 5 min. The cooking loss (CL) was determined by evaporating the combined cooking water and rinse water to dryness at 110 °C and expressed as the percentage of solids loss during cooking. The cooked weight (CW) was calculated as the weight of cooked noodles as a percentage of dry noodle weight before cooking.

### Firmness and tensile strength of GPFRD

The cooked GPFRD was measured for firmness and tensile strength using a texture analyzer (TA-XT Plus, 1125 Stable microsystem Ltd., Surrey, UK) equipped with spaghetti/noodle tensile rig (A/SPR)^20^. Several long strands of noodles were soaked in distilled water for 40 min and cooked to the optimum time. The cooked noodles were rinsed with cool water immediately over a nylon screen and placed in distilled water for testing. After removing the surface moisture from noodle strands with blotting paper, 10 noodle strands were cut to 6.5 cm in length and placed on end into the lower rig arm slot and winding the loosened arm sufficiently, to anchor the noodle end by least two revolutions of the arm. The arm is tightened, and the same procedure is performed to anchor the other noodle end of the upper arm. Tensile strength was tested with rigs arm separation at a speed of 1 mm/s. The initial distance between clamps was set at 10.0 cm. The lower and upper rigs were distanced until the strand ruptured. The maximum stress and breaking distance were recorded. The tensile strength (TS) and breaking distance (BRD) were expressed as the maximum Force before the noodle strand was ruptured (N) and the ratio of the breaking distance to the cross-section area of the noodle strand (mm/mm^2^).

### Consumer acceptance of cooked GPFRD

The participants for the consumer acceptance of the optimized formulation of GPFRD were recruited randomly at Princess Mothers Health Garden, Chiang Mai, Thailand, and written informed consent was obtained from all participants before the experiment was conducted. All participants signed and returned the consent form to the research team, ensured participants consent before participating in the study. The experiment ethics was exempted in this study according to Chiang Mai University’s Institutional Review Board from Chiang Mai University Ethics Committee as all ingredient in GPFRD was assessed for inclined risks toward health issues of any consumers. All methods used in this part of the experiment were carried out following the ethical of sensory evaluation guidelines and regulations^19^. The demographic details of the consumers were not collected as the main concern was the sensory acceptance toward the attribute’s preference of GPFRD as the consumers are all 100% Thai with ranged in age from 18–25 years old. The GPFRD was prepared and put in disposable closed lid plastic cups coded with a three-digit number and evaluate in an individual booth ensuring comfort and privacy. The untrained consumers (n = 100) were asked to rate the preference score for attributes preference (colour, garlic aroma, pepper aroma, and overall aroma) with a 9-point hedonic scale^19^. The product acceptance and purchase intention for the final product was added into the questionnaire on the consumer acceptance test.

### Statistical analysis

All data were analysed in triplicate and reported as a mean±standard deviation. The statistical analysis was conducted using SPSS 11.0 (SPSS Inc., IBM Corp., IL, USA) using Duncan’s multiple range test (DMRT) with a significant level determined at a 95% confidence limit (p < 0.05). The regression analysis on RSM to indicate the optimized ratio of DGP and DWPP was appointed using Design Expert 6.0 (Stat Ease Inc., MN, USA).

## Results and discussion

### Identification of main volatile compounds of GPP using gas chromatography flame ionisation (GC-FID) coupled with gas chromatography olfactometry (GC-O)

The retention of garlic-pepper aroma was detected as beta-pinene (weak to medium), allyl methyl sulfide (medium to strong), dimethyl disulfide (weak to medium), dimethyl trisulfide (weak to strong), limonene (weak), and beta-caryophyllene (weak), respectively. The results showed that the garlic aroma characteristics can be defined from allyl methyl sulfide (garlic), dimethyl disulfide (onion), and dimethyl trisulfide (fried garlic) while the white pepper aroma characteristics can be defined as beta-pinene (pine), limonene (citrus), and beta-caryophyllene (woody). Moreover, the findings of main pepper aroma compounds consisted of beta-pinene, limonene, and beta-caryophyllene^8^ compared to the phytochemical, antioxidant and aroma compounds of *Piper longum* L. and *Piper chaba*. Those can be used as information to confirm the main aroma compounds of GPP^21,22^.

### Physical properties, main volatile compounds and active compounds on GPP

The moisture content, water activity, and colour value (L*, a*, and b*) were a significant difference and was in the range of 6.45% to 7.86%, 0.34 ± 0.01 to 10.36 ± 0.03, 81.56 ± 0.01 to 88.92 ± 0.03, 0.35 ± 0.10 to 0.77 ± 0.02, and 13.95 ± 0.26 to 19.53 ± 0.15, respectively. The results showed significant amount of volatile compounds from GPP as beta-pinene (0.006–0.037 mg/g), allyl methyl sulfide (0.008–0.101 mg/g), dimethyl disulfide (0.001–0.034 mg/g), dimethyl trisulfide (0.194–0.606 mg/g), limonene (0.016–0.114 mg/g), and beta-caryophyllene (0.007–0.226 mg/g). The bioactive compounds of GPP also showed a significant difference amount of allicin (garlic) as 8.45–12.65 mg/g powder, and piperine (white pepper) as 0.04–1.31 mg/g powder (Table 2). The increasing amount of DGP and DWPP caused the volatile compounds and bioactive compounds from GPP to be increased as suggested in the findings from many pieces of research on DGP and DWPP^10,14,16^.

**Table 2:** Main volatile compounds content and bioactive compounds from GPP.

Treatment^1^	Main volatile compounds						Active compounds	
	beta-pinene (mg/g extract)	allyl methyl sulfide (mg/g extract)	dimethyl disulfide (mg/g extract)	dimethyl trisulfide (mg/g extract)	limonene (mg/g extract)	beta-caryophyllene (mg/g extract)	allicin (mg/g sample)	piperine (mg/g sample)
1	0.020 ± 0.001^cd^	0.037 ± 0.002^c^	0.002 ± 0.001^bc^	0.606 ± 0.002^a^	0.022 ± 0.002^de^	0.030 ± 0.001^de^	8.74 ± 0.02^e^	0.22 ± 0.96^e^
2	0.019 ± 0.001^cd^	0.098 ± 0.001^ab^	0.001 ± 0.000^d^	0.194 ± 0.023^d^	0.020 ± 0.000^de^	0.037 ± 0.001 ^f^	11.32 ± 0.01^b^	0.20 ± 0.06 ^f^
3	0.029 ± 0.002^b^	0.036 ± 0.003^c^	0.011 ± 0.008^c^	0.432 ± 0.094^bc^	0.078 ± 0.031^b^	0.105 ± 0.002^b^	8.94 ± 0.10^e^	1.15 ± 0.10^b^
4	0.028 ± 0.002^b^	0.084 ± 0.006^ab^	0.012 ± 0.008^bc^	0.489 ± 0.015^abc^	0.046 ± 0.002^c^	0.098 ± 0.006^c^	10.22 ± 0.30 ^cd^	1.12 ± 0.10^b^
5	0.025 ± 0.001^bc^	0.008 ± 0.001^e^	0.001 ± 0.000^d^	0.194 ± 0.002^d^	0.029 ± 0.001^cde^	0.037 ± 0.010^e^	8.45 ± 1.23^e^	0.60 ± 0.08^d^
6	0.024 ± 0.002^bc^	0.101 ± 0.006^a^	0.034 ± 0.024^a^	0.384 ± 0.011^c^	0.023 ± 0.001^de^	0.039 ± 0.005^de^	11.82 ± 0.19^ab^	0.67 ± 0.032^d^
7	0.006 ± 0.000^e^	0.061 ± 0.002^bc^	0.011 ± 0.008^bc^	0.453 ± 0.058^bc^	0.016 ± 0.002^e^	0.007 ± 0.001 ^f^	10.00 ± 0.02^c^	0.04 ± 0.03 ^f^
8	0.037 ± 0.003^a^	0.060 ± 0.004^bc^	0.012 ± 0.008^b^	0.508 ± 0.087^ab^	0.114 ± 0.017^a^	0.226 ± 0.012^a^	10.13.±0.04 ^cd^	1.31 ± 0.02^a^
9	0.021 ± 0.002^c^	0.068 ± 0.005^b^	0.027 ± 0.019^bc^	0.367 ± 0.049^c^	0.042 ± 0.019 ^cd^	0.046 ± 0.009^d^	11.02 ± 1.03^bc^	0.78 ± 0.11^c^
10	0.022 ± 0.001^c^	0.067 ± 0.004^b^	0.026 ± 0.019^bc^	0.356 ± 0.038^c^	0.041 ± 0.018 ^cd^	0.045 ± 0.009^d^	12.65 ± 0.20^a^	0.67 ± 0.03^d^

*note: the different of the letter in the same column indicated the significant difference (*p* $<$ 0.05).

^1^ Treatments are in Table 1.

### Sensory acceptance on GPP

The GPP was evaluated for sensory acceptance (n = 60). The result showed a significant difference in colour, garlic aroma, pepper aroma, overall aroma. The sensory rating score of attributes was in the range of 3.5–6.9; colour (5.5–7.1), garlic aroma (4.7–7.2), pepper aroma (4.5–7.3), and overall aroma (3.5–7.4) as shown in Table 1. The GPP sensory scores were affected by the DGP and DWWP, resulting to affect its overall aroma. The DGP with the medium of amount DWPP showed the highest score of overall aroma because the DWPP contained that pleasant aroma toward consumers and it can enhance the aroma of DGP when it was blended together^23,24^.

### The optimisation and validation of GPP

**Figure 1:**
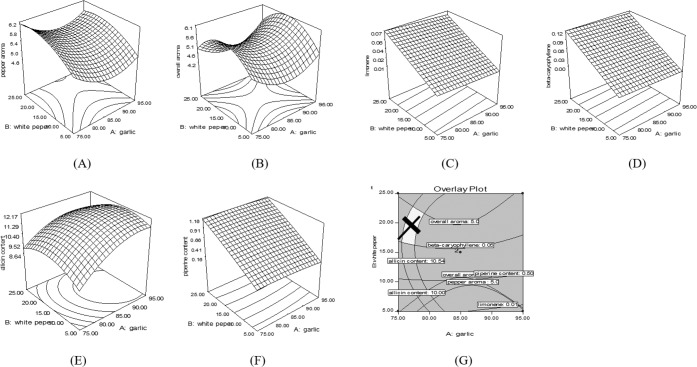
Response surface demonstrated relationship of DGP and DWPP; (**A**) pepper aroma, (**B**) overall aroma, (**C**) limonene, (**D**) beta-caryophyllene content, (**E**) allicin content, (**F**) piperine content, and (**G**) the optimized formula for GPP.

There were five responses (pepper aroma, overall aroma, limonene, allicin, and piperine) that fitted to create the regression equations and can be explained altogether using Table 3 and Fig. 1.

**Table 3:** Regression equation of significant responses from GPP.

Responses	Regression equation	R-square	*p*-value
**Sensory responses**			
pepper aroma	46.19 − 0.98(**A**) + 0.15(**B**) + 0.0056(**A**)^2^ + 0.0036(**B**)^2^	0.8183	0.0429
overall aroma	62.38–1.38(**A**) + 0.20(**B**) + 0.0081(**A**)^2^ − 0.001(**B**)^2^ + 0.001(**AB**)	0.8940	0.044
**Chemical responses**			
beta-caryophyllene	0.009–0.0004(**A**) + 0.006(**B**)	0.6792	0.0187
limonene	0.03–0.0004(**A**) + 0.003(**B**)	0.6785	0.0189
allicin content	–70.20 + 1.73(**A**) + 0.56(**B**) – 0.009(**A**)^2^ – 0.009(**B**)^2^ – 3.25(**AB**)	0.8979	0.0410
piperine content	–1.95 + 0.045(**A**) + 0.055(**B**) – 0.0003(**A**)^2^ – 0.0003(**B**)^2^	0.9931	0.0001

*note: A = DGP and B = DWPP.

The pepper aroma of the treatment 5 (DGP and DWPP at 70 g and 15 g) provided the highest sensory rating score whereas the treatment 2 (DGP and DWPP at 95 g and 5 g) provided the lowest sensory rating scores, resulting from the increasing amount of DGP and DWPP. Figure 1A showed that the increasing of DGP can decrease the sensory score on pepper aroma whereas the increasing of DWPP can increase the sensory score on pepper aroma as the regression equation of pepper aroma showed in Table 3.

The overall aroma of the treatment 5 (DGP and DWPP at 70 g and 15 g provided the highest sensory rating score where the treatment 7 (DGP and DWPP at 85 g and 0.86 g). The increase of DGP affected overall aroma to be decreased whereas the increase of DWPP affected overall aroma to be increased which can be explained in Fig. 1B. These results suggested DGP and DWPP affected the overall aroma of GPP. Besides, the interaction of DGP and DWPP showed an effect on overall aroma, as the increasing of GPP can enhance the overall aroma of the GPP (Table 3). The pepper aroma and overall aroma from GPP found to be affected by DWPP and DGP but in a reverse direction. This is because of the DWPP consist of a volatile compound such as beta-pinene, limonene, and beta-caryophyllene that caused citrusy, green and woody notes and woody, pungent taste in pepper over the garlic that caused sulfurous aroma. Those aromas from the pepper can be found to be a more pleasant perception toward consumers than those aromas from garlic^24^.

The limonene and beta-caryophyllene content from garlic-powder can be expressed in the relationship of DGP and DWPP using linear regression as shown in Table 3. The limonene and beta-caryophyllene content affected by DWPP because of those compounds were the main volatile compounds that can be identified from white pepper^8^. The regression equation of limonene and beta-caryophyllene content showed that DWPP demonstrated a higher effect than DGP. The increase of DWPP affected limonene and beta-caryophyllene content to be increased. That evidence can be found in the same direction from the response surface model as shown in Fig. 1C,D.

The allicin and piperine content were found to be affected by DGP and DWPP as shown in Table 3. The allicin was a bioactive compound that can be found in garlic while the piperine was a bioactive compound that can be found in white pepper^24^. The regression equation of allicin and piperine demonstrated that the increasing amount of DGP affected the allicin content to be increased while the increasing of DWPP affected the piperine content to be increased. The effect of DGP and DWPP can be explained the regression equations more clearly using response surface models as shown in Fig. 1E,F.

The overlay plot of optimised GPP was generated using the high value of sensory rating score (pepper aroma and overall aroma), the volatile compounds (limonene and beta-caryophyllene), and the bioactive compounds (allicin and piperine) as the desirable properties of GPP. The optimised formula for GPP from the RSM consisted of the 78 g garlic powder and the 20 g pepper powder (Fig. 1). The finding of this optimisation was conformed to the investigation on garlic and pepper-flavoured puff snacks which concluded the garlic content and pepper content at 70 g and 18.25 g, respectively^25^. The result suggested that the suitable ratio of garlic and pepper to create acceptance in snacks and noodles was about 4:1. The observation values from the validation of GPP illustrated the highest values of responses as pepper aroma (6.9 ± 0.2), overall aroma (6.6 ± 0.1), limonene content (0.069 ± 0.02 mg/g powder), beta-caryophyllene content (0.101 ± 0.04 mg/g powder), allicin content (10.48 ± 0.18 mg/g powder), and piperine content (0.71 ± 0.11 mg/g powder).

### Physical and texture properties of cooked GPFRD

All GPFRD were cooked using boiling water (98 ± 2 °C) for 1 min. The colour values (L*, a*, b*), tensile, breaking distance, cooking loss, and cooking weight of all samples were significant differences (Table 4). The increasing of GPP affected the lightness to be increased whereas the redness and the yellowness were decreased. This result suggested that the cooked GPFRD become more loosen when engaged with the cooking process using hot water. The higher amount of GPP can be the key factor that rice noodle become less intact because of the fortified powder interrupted the rice starch binding during the extrusion process. The reason that this incident happened because the rice starch partially encapsulated GPP that created flavour encapsulation instead of gelatinization the whole portion of the powder mixture. This occurrence can be explained through the encapsulation in flavour retention during high-temperature short time cooking extrusion process and the extrusion can entrap aroma and flavour compound better as it is a true encapsulation^26^.

**Table 4:** Colour values, texture properties and sensory evaluation of cooked GPFRD.

GPP (%)	Colour value			Tensile (N)	Distance (mm)	Cooking loss (%)	Cooking weight (%)	Sensory evaluation (n = 100)								
	L*	a*	b*					appearance	garlic aroma	pepper aroma	overall aroma	texture	garlic flavour	pepper flavour	overall flavour	overall liking
0	31.27 ± 0.03^d^	3.24 ± 0.03^a^	1.16 ± 0.02^b^	0.20 ± 0.03^a^	27.76 ± 1.5^a^	3.21 ± 0.07^d^	231.11 ± 2.01^c^	4.2 ± 1.7^b^	4.2 ± 0.9^c^	5.4 ± 1.2^b^	5.4 ± 1.2^c^	4.3 ± 2.1^b^	5.4 ± 1.2^b^	5.0 ± 1.3^c^	4.2 ± 1.8^c^	4.5 ± 1.6^b^
2	31.38 ± 0.10^c^	3.02 ± 0.01^b^	1.16 ± 0.02^b^	0.13 ± 0.02^b^	17.76 ± 1.33^b^	4.04 ± 0.12^c^	230.89 ± 3.83^c^	6.8 ± 1.1^a^	6.3 ± 1.2^a^	6.1 ± 1.2^a^	6.1 ± 1.2^a^	6.0 ± 1.5^a^	6.1 ± 1.2^a^	6.8 ± 1.2^a^	6.6 ± 1.2^a^	6.3 ± 1.3^a^
4	32.02 ± 0.02^b^	2.66 ± 0.03^c^	0.76 ± 0.01^a^	0.11 ± 0.01^c^	13.82 ± 0.95^c^	6.49 ± 0.28^b^	256.12 ± 3.26^b^	3.8 ± 1.7^b^	5.0 ± 1.7^b^	4.9 ± 1.6^bc^	5.0 ± 1.7^b^	3.7 ± 1.6^b^	4.9 ± 1.6^b^	5.3 ± 1.5^b^	4.8 ± 1.4^b^	3.9 ± 1.6^b^
6	32.44 ± 0.05^a^	2.29 ± 0.05^d^	0.78 ± 0.02^a^	0.09 ± 0.01^d^	10.68 ± 1.10^d^	8.73 ± 0.13^a^	292.94 ± 3.59^a^	2.7 ± 1.4^c^	4.7 ± 1.9b^c^	4.6 ± 2.0^c^	4.7 ± 1.9^bc^	2.6 ± 1.4^c^	4.6 ± 2.0^c^	4.0 ± 1.8^c^	4.2 ± 1.8^c^	3.0 ± 1.3^c^

*note: the different of the letter in the same column indicated the significant difference (*p* $<$ 0.05).

The decreasing trend of tensile indicated the breaking strength and elasticity of cooked noodles were decreased, which showed that the higher amount of GPP added can cause rice noodles to break easier. In the present study, it was observed that the increase of the GPP affected tensile to be decreased whereas breaking distance, cooking loss, and cooking weight were increased. The high cooking loss indicates high solubility of starch and low cooking tolerance; results in turbid water and a sticky mouthfeel whereas low cooking weight and low tensile cause by poor water-binding capacity^27^.

### Chemical properties of cooked GPFRD

The results exhibited the range of volatile compounds followed the retention time as beta-pinene (1.15–2.96 mg/g), allyl methyl sulfide (0.79–2.83 mg/g), diallyl disulfide (0.44–0.15 mg/g), diallyl trisulfide (0.44–1.15 mg/g), limonene (0.15–0.19 mg/g), and beta-caryophyllene (0.46–0.54 mg/g) (Table 5). The cooked GPFRD also showed a significant difference of allicin and piperine content. The results of allicin content and piperine content were in the range of 35.52–57.60 mg/g and 0.014–0.211 mg/g. The increasing of GPP affected the main volatile compounds, allicin, and piperine to be increased because of the higher ratio of garlic-pepper in the GPP appeared to contain a higher amount of total volatile base value significantly^28^.

**Table 5:** Volatile compounds and active compounds of cooked GPFRD.

GPP (%)	Main volatile compounds						Active compounds	
	beta-pinene (mg/g)	allyl methyl sulfide (mg/g)	dimethyl disulfide (mg/g)	dimethyl trisulfide (mg/g)	limonene (mg/g)	beta-caryophyllene (mg/g)	allicin (mg/g)	piperine (mg/g)
0	—	—	—	—	—	—	—	—
2	1.15 ± 0.02^c^	0.79 ± 0.07^c^	0.35 ± 0.01^b^	0.44 ± 0.05^b^	0.15 ± 0.01^b^	0.46 ± 0.01^b^	35.52 ± 0.93^c^	0.014 ± 0.002^b^
4	2.20 ± 0.05^b^	1.88 ± 0.01^b^	0.44 ± 0.01^b^	1.15 ± 0.05^a^	0.15 ± 0.01^b^	0.52 ± 0.02^a^	46.67 ± 1.25^b^	0.020 ± 0.004^a^
6	2.96 ± 0.01^a^	2.38 ± 0.11^a^	0.64 ± 0.08^a^	1.15 ± 0.02^a^	0.19 ± 0.02^a^	0.54 ± 0.02^a^	57.60 ± 2.22^a^	0.211 ± 0.001^a^

*note: the different of the letter in the same column indicated the significant difference (*p* $<$ 0.05).

### Sensory and consumer acceptance of cooked GPFRD

The cooked GPFRD with 2% GPP provided the highest sensory rating score; appearance (6.8 ± 1.1), colour (6.7 ± 1.0), garlic aroma (6.3 ± 1.2), pepper aroma (6.1 ± 1.2), overall aroma (6.1 ± 1.2), texture (6.0 ± 1.5), garlic flavour (6.1 ± 1.2), pepper flavour (6.8 ± 1.2), overall flavour (6.6 ± 1.2), spiciness (6.1 ± 1.6), aftertaste (6.0 ± 1.6), and overall liking (6.3 ± 1.3) as shown in Table 4 with consumer acceptance at 82.0% and purchase intention at 74.0%. The results revealed that garlic-pepper affected on sensory rating score to be increased from non-added GPP to 2% GPP. The increase of GPP into rice noodles over 2% can cause the sensory rating score to be decreased. The amount of GPP more than 2% was exceeded the degree of acceptability of consumers, which can be exhibited the aroma and flavour too strong toward the consumer. Similarly, some investigations suggested in the sensory tasting of noodles added with moringa leaves powder that only the increased amount of moringa leaves powder to 0.3% can retain the product acceptability from the consumer. This is also suggested that only a small number of herbs and spices should be fortified into food products because only slightly quantity can provide a high volume of aroma and flavour which can be suitable for consumers^16^.

## Conclusion

The findings from this experiment revealed that the GPP exhibited main aroma compounds and its characteristic from both DGP and DWPP as beta-pinene (pine), allyl methyl sulfide (garlic), dimethyl disulfide (onion), dimethyl trisulfide (fried garlic), limonene (citrus), and beta-caryophyllene (woody). The suitable ratio of DGP and DWPP for GPP was 78 g and 20 g, provided the acceptable sensory rating score of pepper aroma (6.9 ± 0.2) and overall aroma (6.6 ± 0.1). This optimum amount also provided the highest amount of limonene (0.069 ± 0.02 mg/g), beta-caryophyllene (0.101 ± 0.04 mg/g), allicin (10.48 ± 0.18 mg/g), and piperine (0.71 ± 0.11 mg/g). Moreover, the GPP at 2% was a suitable amount to produce the most preferable GPFRD using the extrusion process. A suitable amount of GPP can provide the preferable properties of flavoured rice noodles. Besides, this work can provide introductory data for further research on the correlation between volatile compounds, antioxidant compounds, bioactive compounds, and sensory perception.

## Data Availability

The datasets generated during and/or analysed during the current study are available from the corresponding author on reasonable request.
